# Levofloxacin pharmacokinetics in saliva as measured by a mobile microvolume UV spectrophotometer among people treated for rifampicin-resistant TB in Tanzania

**DOI:** 10.1093/jac/dkab057

**Published:** 2021-03-01

**Authors:** Sagal Mohamed, Happiness C Mvungi, Margaretha Sariko, Prakruti Rao, Peter Mbelele, Erwin M Jongedijk, Claudia A J van Winkel, Daan J Touw, Suzanne Stroup, Jan-Willem C Alffenaar, Stellah Mpagama, Scott K Heysell

**Affiliations:** 1 Division of Infectious Diseases and International Health, University of Virginia, Charlottesville, VA, USA; 2 Kibong'oto Infectious Diseases Hospital, Sanya Juu, Tanzania; 3 Kilimanjaro Clinical Research Institute, Moshi, Tanzania; 4 Department of Clinical Pharmacy and Pharmacology, University Medical Center Groningen, University of Groningen, Groningen, The Netherlands; 5 School of Pharmacy, Faculty of Medicine and Health, University of Sydney, Sydney, Australia; 6 Westmead Hospital, Sydney, Australia; 7 Marie Bashir Institute for Infectious Diseases and Biosecurity, University of Sydney, Sydney, Australia

## Abstract

**Background:**

Early detection and correction of low fluoroquinolone exposure may improve treatment of MDR-TB.

**Objectives:**

To explore a recently developed portable, battery-powered, UV spectrophotometer for measuring levofloxacin in saliva of people treated for MDR-TB.

**Methods:**

Patients treated with levofloxacin as part of a regimen for MDR-TB in Northern Tanzania had serum and saliva collected concurrently at 1 and 4 h after 2 weeks of observed levofloxacin administration. Saliva levofloxacin concentrations were quantified in the field via spectrophotometry, while serum was analysed at a regional laboratory using HPLC. A Bayesian population pharmacokinetics model was used to estimate the area under the concentration–time curve (AUC_0–24_). Subtarget exposures of levofloxacin were defined by serum AUC_0–24_ <80 mg·h/L. The study was registered at Clinicaltrials.gov with clinical trial identifier NCT04124055.

**Results:**

Among 45 patients, 11 (25.6%) were women and 16 (37.2%) were living with HIV. Median AUC_0–24_ in serum was 140 (IQR = 102.4–179.09) mg·h/L and median AUC_0–24_ in saliva was 97.10 (IQR = 74.80–121.10) mg·h/L. A positive linear correlation was observed with serum and saliva AUC_0–24_, and a receiver operating characteristic curve constructed to detect serum AUC_0–24_ below 80 mg·h/L demonstrated excellent prediction [AUC 0.80 (95% CI = 0.62–0.94)]. Utilizing a saliva AUC_0–24_ cut-off of 91.6 mg·h/L, the assay was 88.9% sensitive and 69.4% specific in detecting subtarget serum AUC_0–24_ values, including identifying eight of nine patients below target.

**Conclusions:**

Portable UV spectrophotometry as a point-of-care screen for subtarget levofloxacin exposure was feasible. Use for triage to other investigation or personalized dosing strategy should be tested in a randomized study.

## Introduction

The burden of MDR-TB/rifampicin-resistant TB (RR-TB) continues to grow with half a million cases estimated in 2018 and with a global treatment success rate of only 56%.^1^ More favourable outcomes from novel regimens have prompted recent changes to MDR-TB treatment guidelines and later-generation fluoroquinolones (moxifloxacin and levofloxacin) along with bedaquiline and linezolid now form the backbone of MDR/RR-TB therapy.^2^ Fluoroquinolones have not only been associated with an increased likelihood of MDR/RR-TB treatment success, but, compared with other anti-TB drugs, have high bioavailability, ease of dosing, relatively low cost and a limited side effect profile (with levofloxacin favoured over moxifloxacin in many settings due to less potential for prolongation of the QTc interval of the ECG).^3–7^ Nevertheless, fluoroquinolones display significant individual pharmacokinetic variability.^8^^,^^9^ We and others have found that MDR/RR-TB treatment success is correlated with a high serum area under the concentration–time curve relative to the MIC of levofloxacin for the infecting *Mycobacterium tuberculosis* strain (AUC_0–24_/MIC). Yet optimal AUC_0–24_/MIC may not be achieved by current dosage recommendations of 750 to 1000 mg of levofloxacin daily, especially for strains with higher MICs or when MIC testing is unavailable.^10–15^

Fortunately, with linear kinetics and concentration-dependent killing of levofloxacin, suboptimal dosing can be effectively surmounted by increasing dose to improve bactericidal activity and prevent acquired drug resistance.^3^^,^^10^^,^^16^^,^^17^ Levofloxacin pharmacokinetic parameters correlated with MDR/RR-TB treatment failure in a previous study were AUC_0–24_ <80 mg·h/L and *C*_max_ <8 mg/L, and these values likely represent minimum thresholds to target for individualized dosing.^18^ Such personalized dosing based on pharmacokinetic exposure, often termed therapeutic drug monitoring (TDM), is recommended by ATS/CDC/ERS/IDSA guidelines to be considered for many subgroups of patients at risk of treatment failure and as a means to assure pharmacovigilance and the optimal activity of the companion drugs in a bedaquiline-containing regimen to combat against acquired bedaquiline resistance.^8^^,^^19^ The major barriers to providing personalized dose adjustment by TDM for levofloxacin, and for anti-TB drugs in general, has been a lack of laboratory infrastructure, typically MS and/or HPLC in TB endemic settings, and the need to collect serum at multiple timepoints over the 24 h dosing interval to establish AUC_0–24_ or determine the true peak concentration (*C*_max_), with subsequent logistical hurdles of cold storage and shipment. Along with others, we have recently demonstrated that AUC_0–24_ for levofloxacin can be accurately estimated with limited sampling strategies using several distinct timepoints within the dosing interval either by multiple linear regression or a population pharmacokinetics Bayesian approach.^9^^,^^18^^,^^20^

In addition to limited sampling strategies, non-serum alternatives represent the most practical means to deliver TDM for levofloxacin to the point-of-care. Saliva has been postulated as an effective matrix for anti-TB drug concentration assays given that collection is non-invasive and many drugs adequately penetrate salivary tissue.^21^^,^^22^ Levofloxacin distributes to all body compartments and, for example, in lung tissue, levofloxacin concentrations may be 1.5 to 4 times higher than serum concentrations, but, promisingly, concentrations in saliva have been shown to be similar to those in serum.^16^^,^^23^^,^^24^ Furthermore, to bypass the need for measurement of saliva concentrations by HLPC or MS, we have developed a portable, battery-powered, UV spectrophotometer that can be used at the point-of-care immediately after sample collection and preparation.^25^

This study seeks to further investigate the capability of saliva as an alternative matrix to quantify adequate levofloxacin exposure as correlated with target serum parameters among people initiating treatment for MDR/RR-TB with levofloxacin-containing regimens. Importantly, concentrations as measured by UV spectrophotometry and serum concentrations as measured by UV HPLC were determined on site in the TB endemic setting of Northern Tanzania.

## Methods

### Patient selection

All patients were recruited consecutively in July 2019 from Kibong’oto Infectious Diseases Hospital (KIDH), a national referral hospital for MDR-TB treatment located in the Kilimanjaro region of Tanzania. The study protocol was approved by the institutional review boards for human subject research at KIDH (KNCHREC0005), the National Institute for Medical Research (NIMR/HQ/R.8a/Vol.IX/2989) in Tanzania and the University of Virginia (UVA-HSR #21848). The study was registered at Clinicaltrials.gov with clinical trial identifier NCT04124055. All participants provided written informed consent. Inclusion criteria for screening included: (i) current admission at KIDH for pulmonary TB; (ii) age of 18 years or greater; (iii) confirmed rifampicin resistance by sputum Xpert MTB/RIF (Cepheid, Sunnyvale, CA, USA); (iv) no detectable fluoroquinolone resistance by sputum Hain MTBDR*sl* (Hain Lifesciences GmBH, Nehren, Germany); and (v) treated with levofloxacin for a minimum of 2 weeks prior to enrolment. Exclusion criteria were: (i) pregnancy at any gestation or breastfeeding; (ii) comorbidities, such as generalized severe ulcers, Kaposi’s sarcoma and other malignancies; and (iii) inability to provide consent due to critical illness or altered mental status.

Baseline physical and clinical characteristics were recorded for each patient, including age, gender, weight, height, BMI, medical comorbidities (HIV, diabetes and chronic kidney disease) and smoking and alcohol history, as well as all medications they were taking concurrently. Preliminary laboratory testing included creatinine and blood glucose for all patients and CD4 levels for patients living with HIV. All patients living with HIV were prescribed ART with a regimen of abacavir or tenofovir disoproxil fumarate in combination with lamivudine and dolutegravir. Patients were prescribed 750 mg of levofloxacin if weight was below 50 kg or 1000 mg of levofloxacin if weight was 50 kg or above.

### Specimen collection

Saliva and serum samples were both collected at timepoints of 1 and 4 h after 2 weeks of directly observed levofloxacin administration. Serum samples were collected in vacutainer tubes and promptly frozen after centrifugation and stored at −80°C until transportation to the Kilimanjaro Clinical Research Institute in Moshi, Tanzania for analysis. Personnel collecting saliva samples wore an N-95 mask and were gloved to ensure their safety, though risk of aerosolization was likely low. For saliva collection, Salivette™ (Sarstedt, Nümbrecht, Germany) was used. After collection of the saliva the cotton was then inserted into a 5 mL syringe with a membrane filter (≤0.22 μm) and plunger to remove potential bacteria from the specimen and extract saliva.^26^ Laboratory staff processed and extracted salivary samples within the hospital laboratory at KIDH. Saliva extracted was collected into a storage vial kept at room temperature to be immediately analysed with UV spectrophotometer NP80 (Implen GmBH, München, Germany).^26^ Remaining saliva samples were frozen and stored at −20°C.

### Serum HPLC analysis

HPLC analysis followed our similar approach for levofloxacin detection in serum by HPLC.^27^ Briefly, HPLC analysis was performed on a Dionex UltiMate 3000 system (Thermo Fisher, Waltham, MA, USA) equipped with a quaternary pump, a variable wavelength detector set at 290 nm, a refrigerated autosampler set at 10°C and a column compartment set at 30°C. Levofloxacin and the internal standard difloxacin hydrochloride were separated under continuous gradient elution using an Acclaim (120 Å pore size, C18, 5 μm particle size, 150 × 4.6 mm internal diameter) analytical column. The mobile phase consisted of solvent A (0.05 M sodium phosphate, dibasic, buffer, pH 3.5) and solvent B (0.05 M sodium phosphate, dibasic, buffer, pH 3.5, containing 70% acetonitrile in water). The flow rate was set at 0.6 mL/min. The time program for gradient elution was continuous from 15% to 75% of reverse phase, solution B. The total analysis time for each sample was 20 min. Chromatograms were developed with Chromeleon 7.2 software (Thermo Fisher Scientific, Waltham, MA, USA). The nine-point calibration curve ranging from 0.5 to 15 mg/mL was linear with a correlation coefficient of 0.9997. The intra-day and inter-day precision were 1.90–2.44% RSD and 3.30–5.65% RSD, respectively (where RSD stands for relative standard deviation).

### Saliva UV spectrophotometry analysis

In brief, the experiments were performed on a mobile NP80 NanoPhotometer (Implen, München, Germany), a mobile UV/VIS spectrophotometer with a scan range of 200–900 nm, a scan time of 2.5–4 s and a bandwidth of <1.8 nm, with a sample volume of 0.3–2 μL. A small drop of saliva of at least 3 μL was placed on the sample surface of the spectrophotometer with a disposable pipette.

The levofloxacin calibration curve was linear over a range of 2.5–50.0 mg/L with a correlation coefficient of 0.9994. The accuracy ranged from –5.5% to 2.5% and overall precision ranged from 2.1% to 16.1%. For analysis of patient saliva samples, a small drop (<3 μL) of saliva was placed on the sample surface, with the use of a disposable Pasteur pipette. The path length was set at 0.67 mm and a UV/VIS spectrum was scanned in the 200–900 nm range. The smoothing function was turned off.

To increase sensitivity and selectivity, the levofloxacin concentration was quantified by using the amplitude of the second-order spectrum between 300 and 400 nm. The second-order spectrum was calculated using the Savitsky–Golay method.^26^ These calculations were done using a proprietary Excel spreadsheet (Microsoft, Redmond, WA, USA).

### Statistical analysis

Stata 15 (College Station, TX, USA) was used for descriptive statistics and to calculate medians and IQRs for patient demographic characteristics. A Bayesian popPK model (version 3.82; Mediware, The Netherlands) derived from multiple cohorts of patients treated with levofloxacin for MDR/RR-TB, including a similar cohort of Tanzanian patients (and with built-in parameters, such as age, sex, renal function and levofloxacin dosage), was used to estimate AUC_0–24_.^20^ The normal distribution of data was ascertained by visual inspection of boxplots. A non-parametric Mann–Whitney *U*-test was used to assess the differences between serum and saliva AUC_0–24_ between subgroups. Passing–Bablok regression was conducted to assess for methodological agreement between levofloxacin concentrations in saliva and serum, using R software (version 4.0.1, http://r-project.org). MIC testing for levofloxacin of individual *M. tuberculosis* strains was not performed in this study; however, in a recent study of 124 isolates from Tanzanian MDR-TB patients initiating therapy at KIDH, we found the median MIC of levofloxacin to be 0.5 mg/L (Scott K. Heysell, Stellah Mpagama and Margaretha Sariko, unpublished data; NCT03559582). Thus, using the AUC_0–24_/MIC target of 160 associated with microbiological cure for pulmonary TB in a hollow-fibre model and replicated in a cohort with MDR-TB from KIDH,^18^ and the expected population median MIC of 0.5 mg/L, we set the target serum AUC_0–24_ at 80 mg·h/L. Area under the receiver operating characteristic (ROC) curve and 95% CI were calculated using the pROC package,^28^ to detect serum AUC_0–24_ <80 mg·h/L, the parameter corresponding to inadequate exposure and higher likelihood of treatment failure, and which would trigger dose increase.

## Results

Fifty-one patients were enrolled with one person unable to produce an adequate saliva specimen and five others for whom regimens did not contain levofloxacin at the time of scheduled sample collection. Forty-five patients with MDR-TB and both serum and saliva collection were ultimately included in the study, 11 (25.6%) of whom were women, 16 (37.2%) of whom were living with HIV and only 2 (4.7%) of whom were identified as having diabetes (Table 1). All people living with HIV were taking ART at the time of serum and saliva collection. Saliva collection was found to be convenient and comfortable for the patients and proceeded without complications. Compared with venipuncture for serum collection and centrifugation, sample collection proved efficient for nursing staff with minimal interruption in workflow.

**Table 1.** Participant characteristics; *N = *45

Characteristic	*n* (%) or median (IQR)
Female	11 (25.6)
Age (years)	39 (32–45.5)
Weight (kg)	51 (46.5–61)
Height (cm)	168 (163–173)
BMI (kg/m^2^)	18.8 (16.4–21.3)
Prior history of TB	21 (48.8)
HIV positive	16 (37.2)
Diabetes mellitus	2 (4.7)
Levofloxacin dose (mg/kg)	15.38 (13.8–16.6)
Creatinine (mg/dL)	1.05 (0.9–1.3)
Other anti-TB medications	
clofazimine	35 (77.7)
bedaquiline	34 (76)
pyrazinamide	32 (71.1)
ethionamide	22 (48.9)
linezolid	20 (44.4)
*p*-aminosalicylic acid	9 (20)

Median *C*_max_ values for levofloxacin were higher in serum (14.4 mg/L, IQR = 9.8–16.4) compared with saliva (10.9 mg/L, IQR = 8.1–14.1) (*P *=* *0.21), as were estimated AUC_0–24_ values in serum (140 mg·h/L, IQR = 102.4–179.1) compared with saliva (97.1 mg·h/L, IQR = 74.8–121.1) (*P *=* *0.001) (Table 2). Inter-individual variation was higher for saliva than serum as demonstrated by the coefficients of variation (Table 2). No significant differences were observed in estimated AUC_0–24_ in saliva or serum between subgroups of sex, HIV status and those with normal (creatinine ≤1.5 mg/dL) and abnormal renal function.

**Table 2.** Levofloxacin concentrations and pharmacokinetic parameters in saliva and serum

Pharmacokinetic parameter	Serum			Saliva		
	median	IQR	%CV	median	IQR	%CV
Concentration at 1 h (mg/L)	10.7	6.5–15	46	8.8	4.7–13.4	76.9
Concentration at 4 h (mg/L)	11.6	9.2–15.5	49	8.6	6.7–10.5	61.2
*C*_max_ (mg/L)	14.4	9.8–16.4	40.2	10.9	8.1–14.1	61.2
AUC_0–24_ (mg·h/L)	140	102.4–179.1	49	97.1	74.8–121.1	64

Data for pharmacokinetic parameters are presented as medians with IQRs for serum and saliva from 45 patients. *C*_max_ is the maximum concentration of levofloxacin and AUC_0–24_ is the area under the concentration–time curve from 0 to 24 h. Percentage coefficient of variation (%CV) is calculated from mean and SD values as has been reported in other pharmacokinetic comparisons.^12^

**Figure 1.**
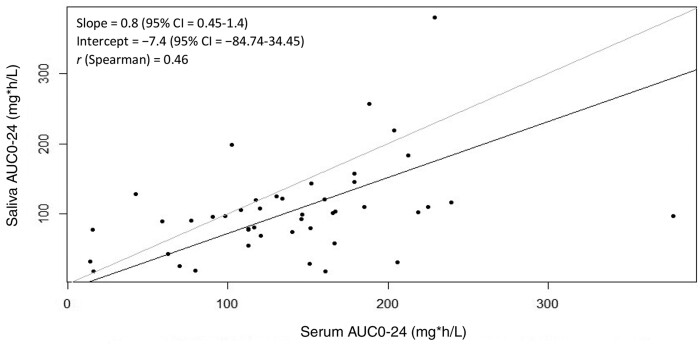
Passing–Bablok analysis of serum and saliva AUC_0–24_. The solid black line represents the fitted Passing–Bablok line and the solid grey line represents the line of identity.

**Figure 2.**
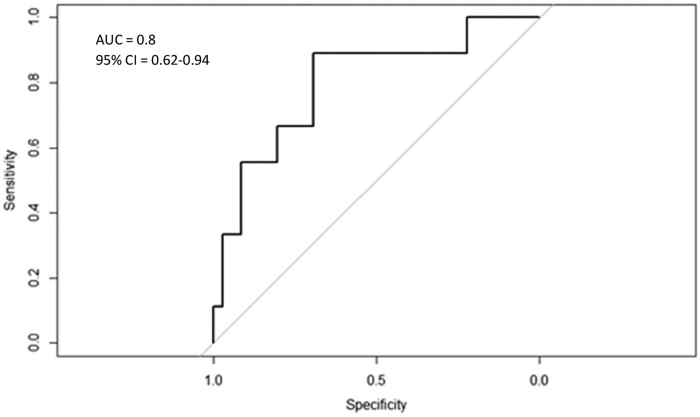
ROC curve to identify serum AUC_0–24_ below 80 mg·h/L.

A modest positive correlation was observed between serum and saliva AUC_0–24_ [*r* (Spearman)  = 0.46, *P *=* *0.001]. Furthermore, in Passing–Bablok analysis (Figure 1), the fitted Passing–Bablok regression line was near to the line of identity (*x *=* y*), with a slope of 0.8 (95% CI = 0.45–1.4) and an intercept of −7.4 (95% CI = −84.74–34.45). The 95% CI range included 1 for slope and 0 for intercept, thereby satisfying the conditions for the line of identity. To further test for validity, an ROC curve constructed to detect serum AUC_0–24_ below 80 mg·h/L resulted in an excellent area under the ROC curve of 0.80 (95% CI = 0.62–0.94) as shown in Figure 2. At a saliva AUC_0–24_ cut-off of 91.6 mg·h/L, the assay was 88.9% sensitive and 69.4% specific in detecting serum AUC_0–24_ values below 80 mg·h/L. At this cut-off, the assay successfully detected 8 of 9 patients below the serum target, but incorrectly identified 11 of 19 as subtarget where serum AUC_0–24_ was above 80 mg·h/L.

## Discussion

Levofloxacin drug concentrations and estimated total pharmacokinetic exposure within a dosing interval were measured for the first time (to the best of our knowledge) from saliva by a spectrophotometer among people treated for MDR-TB in Tanzania. While levofloxacin peak (*C*_max_) and estimated total exposure (AUC_0–24_) were consistently lower in saliva than serum, a positive linear correlation in values was observed, such that saliva exposure can predict serum exposure below clinically relevant thresholds that would either prompt dose adjustment pending optimization of specificity or serve as a screening test to identify those in need of further confirmatory testing with serum assays.^29^^,^^30^ Given that the spectrophotometer did not require the laboratory infrastructure of chromatography or MS instruments, and saliva collection obviated the need for venipuncture, centrifugation and cold transport, this form of personalized care may be suited for decentralized MDR-TB management.

The differences in assayed levofloxacin absorbance by spectrophotometric principles in saliva compared with measured values in serum are expected and the differences largely reflect the unbound fraction from serum that passively diffuses into saliva and, given the inter-individual variability in protein binding, there will consequently be larger variations in salivary concentrations.^31^ Other untested factors that may drive inter-individual variability in salivary concentrations include pH and salivary flow rate.^32^ Despite not measuring salivary pH or unbound levofloxacin concentrations that may have explained the degree of variability, our findings were consistent with the comparison of levofloxacin concentrations in saliva and plasma as measured by LC-MS/MS from a smaller cohort of people with MDR-TB and without HIV in Nepal.^24^ In that cohort, saliva and plasma were collected at timepoints of 0, 1, 2, 4 and 8 h related to dosing, thus resulting in presumably more accurate estimates of AUC_0–24_ than this cohort from Tanzania, where samples were collected at 1 and 4 h only, yet the inter-individual coefficients of variation for saliva concentrations were similarly high from the Nepal cohort.^24^ Further strategies to reduce salivary variability should explore the need for a pH adjustment factor or standardizing patient hydration status, as saliva content is >97% water, and hydration status may influence parotid salivary flow rates and resultant drug concentrations.

Despite the relatively moderate correlation of individual saliva and serum AUC_0–24_, the ROC curve demonstrated adequate prediction of subtarget serum exposure (selected as AUC_0–24_ below 80 mg·h/L), whereby a saliva exposure threshold could be set to maximize sensitivity (not miss subtarget serum exposure) at the cost of only moderate specificity. Thus, even in the absence of assay optimization, we envision a role for levofloxacin salivary concentration testing by spectrophotometry where people with MDR-TB starting treatment in decentralized, community-based settings can be screened for subtarget levofloxacin exposure, with those who screen as potentially low can be triaged for more accurate blood based testing and processing at a more centralized laboratory.^30^ The secondary or confirmatory testing can also be facilitated by collection of dried blood spots instead of serum and while necessitating analysis by chromatography or MS does bypass the need for cold storage and shipment.^33^

Not only do the later-generation fluoroquinolones (levofloxacin and moxifloxacin) remain one of the most important drug classes in MDR/RR-TB therapeutics, as their inclusion in regimens has been associated with treatment success, with subtarget serum exposures having been correlated with worse outcomes, adequate serum exposures as screened for by saliva spectrophotometry or other platforms for therapeutic drug monitoring may be the best means to assure the activity of the background regimen to preserve pharmacovigilance around bedaquiline, arguably the key drug in all oral shorter-course regimens for MDR/RR-TB.^5–7^^,^^34^ Anti-TB drug concentration testing in saliva complements other non-invasive tests, such as colorimetric assays recently developed in urine, which may expand access to personalized dosing not only for those people with TB distant from referral laboratories but also for populations such as children and/or those severely malnourished or with poor venous access where multiple blood draws will be relatively contraindicated or technically difficult.^35^^,^^36^

In addition to the limitations previously mentioned, saliva samples were collected using Salivette™, which may introduce variability in recovery of levofloxacin, but in this study we performed rigorous staff training for standardization of saliva collection procedures and compressed the cotton swab in a syringe through a membrane filter.^26^ One of the potential limitations of the assay is interference with concomitant pyrazinamide at levofloxacin trough concentrations, but not at peak concentrations.^25^ The impact of this analytical limitation was likely negligible as the portion of the studied population taking pyrazinamide was small and we selected samples at 1 and 4 h after drug intake to avoid low levofloxacin concentrations. Lastly, although in previous studies salivary and serum concentrations of levofloxacin were found to be similar, there was only modest correlation in this study, which merits further research into optimal collection points for saliva sampling.^16^^,^^23^^,^^24^

In summary, in this proof-of-concept study, a UV spectrophotometer with adjustments for derivative spectroscopy was successfully utilized to determine levofloxacin concentration in saliva and estimate those treated for MDR-TB with subtarget serum exposures.^25^ Use of a non-invasive matrix, such as saliva, and an inexpensive, battery-powered, portable device, such as a spectrophotometer, allowed sample collection and analytics to be performed on site. Although further studies may be required to understand salivary pharmacokinetic variability and optimize assay specificity, the current performance of the assay is sufficient to be trialled as a screening tool to identify patients likely to benefit from more personalized dosing.

## Funding

This project was financially supported by the Bill & Melinda Gates Foundation, Grand Challenges Program (OPP1191221) and part of the work was supported by the National Institutes of Health (R01 DA044137). S.M. was supported by the National Institutes of Health training grant 5T32 A1007046.

## Transparency declarations

None to declare.
